# miRNAs as potential biomarkers in early breast cancer detection following mammography

**DOI:** 10.1186/s13578-016-0071-0

**Published:** 2016-01-26

**Authors:** Sidney W. Fu, Woojin Lee, Caitrin Coffey, Alexa Lean, Xiaoling Wu, Xiaohui Tan, Yan-gao Man, Rachel F. Brem

**Affiliations:** Department of Medicine (Division of Genomic Medicine), and Department of Microbiology, Immunology and Tropical Medicine, The George Washington University School of Medicine and Health Sciences, 2300 Eye Street, N.W. Ross Hall 402C, Washington, DC 20037 USA; Department of Gastroenterology, Chengdu Military General Hospital, Chengdu, China; Research Lab and International Collaboration, Bon Secours Cancer Institute, Bon Secours Health System, 5801 Bremo Road, Richmond, VA 23226 USA; Department of Radiology, The George Washington University School of Medicine and Health Sciences, Washington, DC USA

## Abstract

Breast cancer is the most common cancer among American women, except for skin cancers. About 12 % women in the United States will develop invasive breast cancer during their lifetime. Currently one of the most accepted model/theories is that ductal breast cancer (most common type of breast cancer) follows a linear progression: from normal breast epithelial cells to ductal hyperplasia to atypical ductal hyperplasia (ADH) to ductal carcinoma in situ (DCIS), and finally to invasive ductal carcinoma (IDC). Distinguishing pure ADH diagnosis from DCIS and/or IDC on mammography, and even combined with follow-up core needle biopsy (CNB) is still a challenge. Therefore subsequent surgical excision cannot be avoided to make a definitive diagnosis. MicroRNAs (miRNAs) are a highly abundant class of endogenous non-coding RNAs, which contribute to cancer initiation and progression, and are differentially expressed between normal and cancer tissues. They can function as either tumor suppressors or oncogenes. With accumulating evidence of the role of miRNAs in breast cancer progression, including our own studies, we sought to summarize the nature of early breast lesions and the potential use of miRNA molecules as biomarkers in early breast cancer detection. In particular, miRNA biomarkers may potentially serve as a companion tool following mammography screening and CNB. In the long-term, a better understanding of the molecular mechanisms underlying the miRNA signatures associated with breast cancer development could potentially result in the development of novel strategies for disease prevention and therapy.

## Background

Breast cancer is one of the most commonly occurring cancers among American women and is the second leading cause of cancer-related deaths. Approximately 1 in 8 American women (12 %) will develop invasive breast cancer in their lifetime, and it is estimated that there will be 231,840 new cases of invasive cancer, 60,290 new cases of noninvasive, in situ cancer, and 40,290 deaths resulting from breast cancer in 2015 [1]. With the current focus on early detection and increased utilization of mammography, more and more non-malignant lesions, such as atypical ductal hyperplasia (ADH), ductal carcinoma in situ (DCIS), atypical lobular hyperplasia (ALH), and lobular carcinoma in situ (LCIS), are detected. These lesions are considered one of the most significant risk factors for developing invasive carcinoma [1].

Although the death rates from breast cancer continue to decline as a result of the increased utilization of mammography, it is not a definitive early screening tool due to its limited sensitivity and specificity [2]. Fayanju et al. reported that about 40 % of the 9000 registrants surveyed refused to use mammography due to “fears of cost,” while another 13 % refused to use it due to “mammogram-related pain [3].”

Over the past decade, research has largely shifted focus from mRNA biomarkers to microRNAs (miRNAs) as a new potential screening biomarker for breast cancer. In numerous studies, including our own, researchers have found miRNAs to be aberrantly expressed in tissue and serum/plasma in patients with breast cancer [4–6]. Although the role of miRNAs in carcinogenesis is unclear, evidence suggests that miRNAs are involved in the initial development and progression of breast cancer by modulating the expression of their target proto-oncogenes or tumor suppressor genes (TSG) at the posttranscriptional level [4]. We, as well as others (FFPE and 638 paper), have identified numerous miRNAs that are aberrantly expressed during breast cancer progression, indicating that miRNAs may serve as a new noninvasive, cost-effective screening tool, as well as a companion tool to be used in conjunction with mammography for more accurate and specific diagnoses [5, 6]. Furthermore, there are now ongoing studies on differential miRNA expression among different pathological stages of breast leisons: ADH, DCIS and IDC [6].

To distinguish pure ADH diagnosis from advanced lesions, such as DCIS and/or IDC following a mammography, and even combined with follow-up core needle biopsy CNB is still a challenge. In this review, we summarized the nature of early breast lesions and the potential use of miRNA molecules as biomarkers in early breast cancer detection. In particular, miRNA biomarkers may potentially serve as a companion tool following mammography screening and CNB. In the long-term, a better understanding of the molecular mechanisms underlying the miRNA signatures associated with breast cancer development could potentially result in the development of novel strategies for disease prevention and therapy.

### Linear model of breast cancer progression

The relationship between ADH, DCIS, and invasive cancer is not fully understood. Historically, breast cancer was viewed as a progression from normal epithelial cells to hyperplasia (with or without atypia), to in situ carcinoma, eventually resulting in invasive carcinoma and metastasis. The most widely accepted model of breast cancer development at the present time is that ductal cells undergo a neoplastic transformation which starts from normal epithelium to flat epithelial atypia (FEA), evolving to atypical ductal hyperplasia (ADH), into ductal carcinoma in situ (DCIS), and finally, into invasive ductal carcinoma (IDC). This model is supported by genomic and immunohistochemical data which shows distinct features at each stage in development [7]. The current understanding of breast cancer development supports this model, placing invasive cancer at the end of a multi-stage transition from normal breast epithelium; however, recent discoveries in molecular genetics and immunohistochemistry have highlighted the complexity of the process, identifying many different and divergent pathways resulting in invasive cancer. Cancer cells undergo their characteristic changes over a significant timeframe, as genes are activated and inactivated in a series of steps [8]. Over the course of tumorigenesis, these various cellular and genetic changes present as pathologically distinct entities.

Via a model of continuation, including FEA, ADH, DCIS, and IDC, is supported by morphological, epidemiological, and immunohistochemical data, as well as genomic and transcriptomic studies. Patterns of genomic changes in DCIS, which parallel those occurring in IDC, have been previously discussed. FEA and ADH also demonstrate similar distinct genetic changes, which overlap with some of those of low-grade DCIS [7]. Notably, the current evidence supports ADH as the precursor for low-grade, but not high-grade, DCIS; the latter which is believed to have a distinct progression, which may include adenosis. Furthermore, there is evidence for progressive allelic damage from stages of ADH to DCIS and finally IDC [8].

The current clinical approach used to prevent breast cancer involves an attempt to diagnose at the earliest possible time, which is the most easily treatable stage in order to maximize the chances for positive outcomes. Thus, improving our understanding of early stage cancers, as well as pre-neoplastic processes in the breast, is crucial to improving future therapy. ADH represents a crucial pathological stage in the transition from what is considered normal to what is considered cancerous in ductal cells. Ductal subtype accounts for 40–75 % of diagnosed breast cancer cases [7]. We are currently aware of many of the molecular biomarkers that may be indicative of a development from normal breast epithelium to ADH, which will be discussed in detail below. Despite our current capabilities, it is important to recognize that ADH is not an obligate precursor to in situ or invasive cancer, as noted by Kuerer, as only 14–50 % of women with ADH or DCIS will develop invasive cancer in their lifetime if left untreated [9]. Additionally, there have been studies that show the changes of molecular markers that occur during the DCIS stage actually contribute to the invasive characteristics of cancer. Therefore, continued efforts to understand the linear view of breast cancer progression and, in particular, what causes this development from normal, to premalignant, to malignant stages will offer necessary information in efforts to develop new effective treatments.

### Distinction between ADH and DCIS

It is important to recognize the differences between ADH and DCIS, as they are both noninvasive lesions with several overlapping characteristics. ADH is a benign breast condition characterized pathologically by some, but not all. ADH is seen in 1–9 % of core needle biopsies [10], but is clinically noteworthy as a marker for risk of future breast cancer development, and for its frequent association with DCIS and invasive carcinoma upon surgical excision. ADH is now considered a non-obligate precursor for low-grade DCIS; in fact, the distinction between ADH and DCIS is often unclear, causing diagnosis to vary among institutions.

Although the distinction between ADH and DCIS is unclear, DCIS is often categorized as non-invasive breast cancer. Histologically, DCIS shows disorganized ductal cells as opposed to ADH, which exhibits normal duct cells with plugged lumens. The ADH is usually defined as a mass less than 2 mm in size, while DCIS is greater than 2 mm. Additionally, low-grade DCIS is defined as a mass that includes two or more breast ducts [7]. Thus, DCIS is often categorized as the following stage of ADH. If DCIS is not surgically removed, it carries a higher risk of becoming invasive cancer than ADH. DCIS also has a higher rate of recurrence, at 30 % within the 5–10 years after initial diagnosis [8].

### Pathological findings

Mammographic screening, which has resulted in a reduction in mortality rates due to breast cancer, has also led to increased diagnosis of benign, non-palpable breast lesions, including atypical, high-risk lesions [11]. ADH, for instance, is diagnosed in the breast most often via core needle biopsy or surgical excision. Historically, ADH was a diagnosis of exclusion; the diagnosis was reserved for lesions whose cells resembled low-grade DCIS but did not fulfill all criteria for DCIS [12]. Though the criteria for a diagnosis of ADH have been refined, histopathological distinctions between ADH and some low-grade DCIS remains problematic. Diagnosis is primarily quantitative, as cellular appearance is similar. Distinction between ADH and DCIS is based on size and involvement, as mentioned above.

Current diagnosis of ADH involves examination of three variables: architectural pattern, cytology, and disease extent [13]. The cells of ADH are small to medium sized, round, cuboidal, or polygonal shaped, regularly arranged, and hyper-chromatic with evenly distributed nuclei and only small, singular nucleoli. Small foci of necrosis are uncommon, yet may be present and do not necessarily indicate DCIS [13]. Monotonous cells, uniformly spaced with rigid secondary spaces, and low-grade nuclei, characterize cells of both ADH and DCIS. Regardless of lesion size, partial involvement of spaces supports a diagnosis of ADH. The diagnosis of DCIS is generally only made when a lesion meets these criteria: either a diameter greater than 2 mm or more than two separate duct spaces, as these have been associated with increased risk for breast cancer [14].

It has been noted that ADH is thought to lie on a histopathological spectrum between usual hyperplasia (lacking atypia) and low-grade DCIS. Usual hyperplasia differs in appearance from ADH in variability of cells, indistinct cell borders, and nuclear overlap, with irregular secondary spaces [15].

Due to the similarities in cellular appearance, particularly between ADH and low-grade DCIS, as well as the small size of cores resulting from minimally invasive breast biopsy, pathological diagnosis remains complicated. In fact, accurate diagnosis of ADH requires morphological, histological, and architectural size criteria of a lesion, which may be interrupted by core biopsy placement [13]. For this reason diagnosis is not always reproducible between pathologists, and many argue that accurate diagnosis of ADH can only be made from excisional biopsy. In a study involving six experienced pathologists who reviewed cases of usual ductal hyperplasia (UDH), ADH, and DCIS, all six were able to reach consensus on only 58 % of the cases [15]. There has, to date, been no biomarker identified that is more useful than histopathologic diagnosis, though epigenetic changes associated with ADH and DCIS have been the subject of much recent research (see below “Biological findings” section) and may aid in the development of a reliable diagnosis in the future.

### Clinical implications

The advent of population-based mammography screening has led to the increased detection of invasive breast cancers as well as a larger number of non-invasive cancer precursors, such as DCIS, and non-cancerous, high-risk lesions such as ADH. Though some consider ADH to be associated with over diagnosis, clinical diagnosis of ADH has two important implications:

(1) In approximately 20–50 % of ADH cases an immediately adjacent cancer is found upon surgical excision, and as a result excision is recommended for all cases [16]. (2) Diagnosis of ADH is associated with a 4–5 times increased risk of development of breast cancer at a median follow up of 17 years, a risk which is increased to 8–10 times as likely in women whose family history includes a primary relative with breast cancer [17].

Risk assessment is important as it includes a need for vigilant annual mammographic screenings and requires annual breast MRIs; these preventative measures may include the consideration of utilizing chemopreventative agents such as Tamoxifen, which have been shown to reduce the risk of developing breast cancer. Additionally, the National Surgical Adjuvant Breast and Bowel Project found that Tamoxifen can reduce risk of breast cancer by approximately 50 % in high-risk women, and has showed an astounding 86 % decrease in incidents of breast cancer among women with a history of ADH. These findings support the importance of reliable ADH diagnosis procedures, as the utilization of chemoprophylaxis selective estrogen reuptake modulators (SERMs) to treat breast cancer may be an effective tool in reducing mortality [18].

In the case of a suspicious lesion sampled by CNB which results in a diagnosis of ADH, standard follow-up care is currently the surgical excision of the lesion for all patients, due to risk of associated cancer foci. Adjacent breast cancer is found in 18–48 % of cases with a 14-gauge needle and 19–25 % with an 11-gauge needle, with some estimates even higher [10]. Unfortunately, this practice subjects patients to the risks associated with surgery, discomfort, and what may be unnecessary cost, as the majority patients with ADH do not have an associated carcinoma and many will not develop breast cancer in their lifetime.

Current difficulties in the clinical management of ADH are the inability to reliably assess risk, or the presence of an adjacent cancer, and to identify prospective patients who may not require surgical excision. Many studies have been conducted to identify factors associated with upgrade to cancer upon excision, and, although predictors have been identified, none are yet considered reliable enough to justify forgoing treatment for an ADH patient. Generally, ADH found in more than two foci is a significantly more reliable predictor of DCIS upon excision than when found in less than two foci. In one study, 39.0 % of cases (16/41) of ADH in more than two foci versus 7.3 % with ADH in less than two foci had an associated DCIS. In the same study, the lowest risk group identified, with no associated DCIS, exhibits one or two foci of ADH, micro-calcifications in the lesion, and all calcification removed by biopsy (confirmed by post-biopsy imaging) [17]. Many clinics have been able to identify lower-risk subtypes of ADH that may have a risk of associated DCIS of less than 2 %, which is the same criterion that indicates a need for imaging follow-up for BIRADS-3 lesions [19]. However, the upgrade rates remain too high to support only surveillance of these atypical lesions, as a breast imaging-reporting and data system (BIRADS) score of B3 would indicate [13]. Further research aimed at identifying reliable predictors of breast cancer risk in ADH patients is needed in order to improve the efficiency of therapeutic recommendations, as well as to minimize anxiety and procedural risks.

Finally, it is important to mention that though the evidence supports surgical excision of every ADH lesion detected by CNB due to its frequent association with carcinoma, atypia found on an excision margin is not considered a risk factor for cancer recurrence, and as a result, in these cases further surgery is not currently recommended [12].

### Biological/molecular findings

Techniques used for classifying ADH and DCIS are not always reproducible between centers, and because of this, there have been replicated studies aimed at finding stable biological and molecular markers for diagnosing ADH and DCIS. In two of the studies, done by O’ Connell et al. and Arpino et al. respectively, 45 % of ADH patients and 77 % of DCIS patients share the loss of heterozygosity (LOH) in chromosome 16p and 17q with ipsilateral breast cancer when harvested from the same breast. However, the percentage drops to 42 % in ADH and 70 % in DCIS when harvested from non-invasive breast. The rate of LOH was low at individual loci in ADH, which suggests that these individual lesions may be genetically heterogeneous. Furthermore, LOH was more common in DCIS than in regular hyperplasia, which further indicates that DCIS is a more advanced stage in malignant evolution [20]. Because LOH has been identified in ADH, DCIS and IDC lesions with similar frequency in a study done by Arpino et al., this supports the idea that ADH is more of a clonal lesion and belongs in the same spectrum as in situ carcinoma [17].

Another study done, by Ma et al., compared laser-microdissected samples of ADH, DCIS, and IDC at the transcriptome level. This study found that the samples from these three stages are very similar in terms of transcriptome level, and that they may derive from the same clonal origin, further supporting the claim that ADH is a precursor lesion to DCIS. The study also found several genetic alterations that occur in the ADH cells that persist through the DCIS and IDC stages; this analysis, however, did not identify the genetic changes that are distinct to each pathological phase of breast cancer, suggesting heterogeneity [19].

### miRNA as the new biological/molecular marker

Although breast cancer research using genetic markers has showed ADH to be a definite genetic precursor to DCIS and IDC, it has also proved to be difficult in distinguishing one pathological phase of breast lesion from another. Recent years, there have been many studies that have found miRNA expression dysregulation between normal, ADH, DCIS and IDC (Tables 1 and 2) [5]. Additionally, the expression of miRNAs can be measured via tissue, plasma, and serum. miRNAs are physiologically functional in regulating oncogene or tumor-suppressor genes (TSG), and thus, down-regulated or up-regulated miRNAs can influence the activity of oncogene or TSG, which in turn can affect tumorigenesis [5].

**Table 1:** Serum, Tissue, and Plasma miRNA expression changes in resection samples in breast cancer

Sample type	Cancer vs. normal	Expression	DCIS/ IDC vs. normal	Expression	References
Serum	miR-451	Down	miR-155	Up	[4]
	miR-148a	Down			[4]
	miR-27a	Down			[4]
	miR-30b	Down			[4]
	miR-182	Up			[21]
	miR-155	Down	miR-19a	Up	[22]
	miR-181b	Down			[22]
	miR-24	Down			[22]
	miR-15a	Up			[23]
	miR-107	Up			[23]
	miR-425	Up			[23]
	miR-139-5p	Down			[23]
	miR-143	Down			[23]
	miR-365	Down	miR-181b	Up	[23]
	miR-155	Up			[23]
	miR-1	Up			[23]
	miR-133b	Up			[23]
	miR-92a	Up			[23]
	miR-18a	Up			[23]
	miR-145a	Down			[23]
	miR-29-b2	up			[24]
	miR-155	Up	miR-24	Up	[24]
	miR-197	Up			[24]
	miR-205	Up			
	miR-195	Down			[22, 25]
	miR-205	Down			[22, 25]
Plasma	miR-148b	Up	miR-571	Down	[26, 27]
			miR-376c	Up	
			miR-139-3p	Down	
	miR-376c	Up	miR-801	Up	
			miR-206	Down	
			miR-193a-3p	Down	
			miR-424	Up	
	miR-409-3p	Up	miR-184	Up	
			miR-409-3p	Up	
			miR-376a	Up	
			miR-526b	Down	
	miR-92a	Up	miR-519a	Down	
			miR-148b	Up	
			miR-190	Up	
			miR-127-3p	Up	
Serum + tissue	miR-132-5p	Down			[28]
	miR-125b-1-3p	Down			
	miR-34c-5p	Down			
	miR-382-3p	Down			
	miR-485-5p	Down			
	miR-323b-3p	Down			
	miR-598-3p	Down			
	miR-224-5p	Up			
	miR-1246	Up			
	miR-184	Up			
Plasma + tissue	miR-16	Up			[27]
	miR-27a	Up			[27]
	miR-150	Up			[27]
	miR-191	Up			[27]
	miR-200c	Up			[27]
	miR-210	Up			[27]
	miR-451	Up			[27]
	miR-145	Down			[23, 27]
	miR-21	Up			[23, 29]
	miR-145	Down			[23]
Tissue	miR-221	Down	miR-10	Down	[30]
			miR-29a	Up	
			miR-21	Up	[6, 30]
			miR-99a	Down	[31]
			miR-151-3p	Up	[31]
			miR-145	Down	[31]
			miR-210	Up	[31]
			miR-10b	Down	[32]
			miR-125b	Down	[32]
			miR-132	Down	[32]
	miR-155	Up	miR-145	Down	[32]
			miR-154-3p	Down	[32]
			miR-382-3p	Down	[32]
			miR-409-3p	Down	[32]
			miR-638	Down	[5, 6]
			miR-200a	Down	[33]
			miR-132	Down	[32]
			miR-638	Down	[5]
			miR-140	Down	[30, 34, 35]
			miR-671-5p	Down	[6]
			miR-183	Up	[6]
			miR-200b	Up	[6]
			miR-200c	Up	[6]
			miR-557	Down	[6]
			miR-1207-5p	Down	[34]
			miR-874	Down	[34]
	miR-30e	Up	miR-556-3p	Up	[34]
			miR-575	Down	[34]
			miR-20a	Up	[34]
			miR-15a	Up	[34]
			miR-1202	Down	[34]
			miR-141	Up	[6, 34]
			miR-19b	Up	[23]
			miR-1925	Down	[23]
			miR-107	Up	[23]
			miR-127-4b	Up	[23]
			miR-1268	Down	[23]
			miR-106b	Up	[23]
			miR-634	Down	[23]

**Table 2:** Tissue miRNA expression changes in resection samples of ADH patients versus normal breast tissue

MiRNA	ADH regulation
miR-1275	Down [34]
miR-638	Down [34]
miR-572	Down [34]
miR-671-5p	Down [34]
miR-183	Up [6]
miR-141	Up [6]
miR-21	Up [6, 34]
miR-200b	Up [6, 34]
miR-200c	Up [6]
miR-15b	Up [23]
miR-183	Up [23]
miR-30d	Up [23]

miRNAs are a class of regulatory RNAs that act to repress the gene expression at the posttranscriptional level. They normally bind to their target mRNAs via base-pairing interactions, which results in either degradation of the target mRNA or inhibition of translation via storage. The miRNAs often bind to the mRNAs within the 3′ untranslated region (UTR) of the target genes [36]. They are involved in various biological processes that are necessary for the maintenance of normal physiological state. In relation to breast cancer, various miRNAs, such as miRNA-200c, are responsible for the regulation of the genetic expression of angiogenesis, apoptosis, proliferation, cell-to-cell adhesion, etc. [37]. Thus, dysregulation of miRNAs expression can lead to dysregulation of cell cycle and growth, which may cause uncontrolled tumor growth [26].

Unlike genetic biological markers, miRNAs hold promise as a future screening marker for breast cancer because they can be measured not only from tissue, but also from serum or plasma. Three separate studies done by Sochor et al., Cuk et al., and Li et al., showed that miR-155, miR-19a, miR-181b and miR-24, which all act to repress TSG, have been up-regulated in DCIS and IDC compared to normal and ADH in serum. They also found that miR-571, miR-206, miR-193a-3p, miR-526b, miR-519a, which all act to repress oncogene, have been down-regulated in ADH, DCIS and IDC compared to normal in plasma (Table 1). Such findings support the idea that the levels of miRNA collected in a patient’s serum or plasma are not only a possible diagnostic tool for breast cancer, but that it can also be a classification tool used to differentiate between ADH, DCIS and IDC if coupled with tissue biopsy as a confirmatory test [22–32].

However, the miRNA expression levels measured from tissue, serum, and plasma are not necessarily consistent. In a study done by Zhu et al., over 174 miRNAs were expressed differently between breast cancer tumors and the normal tissue, but only 109 miRNAs differed in expression level between serum from patients with breast cancer and healthy individuals [28]. Among those miRNAS, only ten were common miRNAs (Table 1). Furthermore, the study found that the change in expression pattern of miRNA between healthy individuals and individuals with breast cancer are opposite in serum and tissue samples in 28 miRNAS [28]. Due to inconsistencies in expression levels of miRNAs in various sample mediums, miRNA biomarkers should be developed tissue-specific.

## Conclusions

With the linear model of breast cancer development, it is accepted that a breast lesion will develop as follows: ADH to DCIS to IDC. With the advent of population screening with mammography, early diagnosis of breast lesions has been possible, however differentiating between the benign ADH and precancerous DCIS or IDC remains illusive. The presence of ADH is known to be a significant factor for the development of breast cancer; however at this time, there are currently no clinical, morphological, or biological markers that can be used to reliably predict whether a premalignant lesion will progress to breast cancer. Using miRNA levels found in serum, plasma, and tissue as a diagnostic and classification tool seems to have a promising future, as there have been hundreds of miRNA identified to play a role in breast cancer. We, as well as other researchers, have shown there are distinct miRNA patterns to distinguish normal breast epithelium from cancerous tissue as well as from DCIS/IDC, and from ADH. Although the consistency of miRNA expression changes among serum, plasma, and tissue samples still need to be proven, it does not preclude the idea that miRNA biomarkers may serve as comparison tool for mammography as a new, inexpensive, non-invasive diagnostic tool in the future.

Patients diagnosed with a lesion on mammography are encouraged to undergo resection, however ADH is not malignant and does not need to be removed.

It is of paramount importance to develop a new, inexpensive screening tool for breast cancer because it will allow wider population, regardless of their socioeconomic status, to receive screening for breast cancer in order to detect breast cancer early, and at the same time to reduce false alarms. Thus, it will also allow for early treatment and a reduction in deaths from breast cancer. Therefore there is great value in utilizing miRNA biomarkers in differentiating ADH from advanced lesions as a potential companion tool following mammography and CNB. In the long-term, an understanding of the molecular mechanisms underlying miRNA biomarkers associated with breast cancer progression could potentially result in the development of novel strategies for disease prevention and therapy.

## Abbreviations

ADH: atypical ductal hyperplasia
DCIS: ductal carcinoma in situ
IDC: invasive ductal carcinoma
miRNAs: microRNAs
CNB: core needle biopsy
